# Comment on: Somatostatin receptor SPECT

**DOI:** 10.1007/s00259-012-2147-3

**Published:** 2012-05-30

**Authors:** Alicja Hubalewska-Dydejczyk, Anna Sowa-Staszczak, Monika Tomaszuk

**Affiliations:** Department of Endocrinology, Jagiellonian University Medical College, ul. Kopernika 17, 31-501 Cracow, Poland

Dear Sir,

In a recent article, Pepe et al. presented a review of the development of compounds for the study of somatostatin receptors and their application in diagnostic imaging [1]. In this very interesting paper the proven effectiveness of labelled somatostatin analogues in the management of patients with various somatostatin receptor-positive diseases is presented (among others its role as a historical turn in the clinical practice and diagnosis of gastroenteropancreatic neuroendocrine tumours, GEP NET). Although we appreciate very much this issue of the *European Journal of Nuclear Medicine and Molecular Imaging* devoted to the most important aspects of the usefulness of somatostatin analogues, we would like to comment on some statements from the review by Pepe et al. In this paper we read: “Given the importance and success of ^99m^Tc as a routine isotope in nuclear medicine imaging, it came as a logical consequence that ^99m^Tc-labelled somatostatin analogues have been developed, namely the ^99m^Tc-N-α-(6-hydrazinonicotinoyl)-octreotide (^99m^Tc-EDDA/HYNIC OCT). At that time propagation of the tracer was hampered by proprietary rights on the peptide. For this reason it has remained a tracer for in-house use.”

To our knowledge ^99m^Tc-EDDA/HYNIC-TOC is the radiopharmaceutical most frequently used for the scintigraphic visualization of sst-positive tumours in Poland. This tracer was registered in Poland in 2004. It is also successfully used in many other European countries according to their national regulations, so calling the availability of this tracer “in-house use” only seems to be disappointing.

^99m^Tc-Labelled radiopharmaceuticals, compared with for example ^111^In-labelled compounds, may improve the quality of images and radiation safety for patients and the staff as a natural consequence of this isotope’s physical properties. As it was stated in another review from this issue by Fani and Maecke ^99m^Tc is the “workhorse” in diagnostic nuclear medicine not without a reason [2]. Also one could read there about the labelling procedure of somatostatin analogues with ^99m^Tc, which may be evaluated as relatively cheap and easily employed in any nuclear medicine hot lab, as well as with high labelling yield and radiochemical purity of the compound assured by the manufacturer and confirmed in our experience.

It was shown that ^99m^Tc-EDDA/HYNIC-TOC reveals high in vitro and in vivo stability, fast background clearance, relatively low accumulation in non-target tissues and rapid detection of somatostatin receptor-positive tumours. Decristoforo et al. and Gabriel et al. studied the imaging abilities of ^99m^Tc-EDDA/HYNIC-TOC compared to Octreoscan [3, 4]. The authors stated that ^99m^Tc-EDDA/HYNIC-TOC was characterized by higher tumour to normal tissue ratios than Octreoscan. A higher target to non-target ratio was also reported by Gabriel et al. for somatostatin receptor scintigraphy (SRS) with the use of ^99m^Tc-N_4_-[Tyr^3^]octreotate (^99m^Tc-Demotate) in comparison also to Octreoscan [5]. ^99m^Tc-EDDA/HYNIC-TOC revealed a higher sensitivity for NET detection than Octreoscan (65.9 vs 51.2 %) [4]. More focal lesions were also detected, especially small liver and lymph nodes metastases (125 vs 90 lesions). This fact was probably related to four times higher ^99m^Tc injected activity in comparison to ^111^In as well as a higher receptor affinity to ^99m^Tc-labelled somatostatin analogues. In the paper by Chrapko et al. [6] about daily clinical practice with the use of ^99m^Tc-EDDA/HYNIC-TOC SRS, the authors concluded that ^99m^Tc-EDDA/HYNIC-TOC SRS is useful in staging as well as in restaging after primary tumour surgery of sst-positive tumours including tumours of unknown primary origin.

Following ^99m^Tc-EDDA/HYNIC-TOC, the Tyr^3^-octreotate (TATE) somatostatin analogue was introduced. It is also octreopeptide and differs from the Tyr^3^-octreotide (TOC) in that the terminal threonine replaces threoninol. The terminal threonine results in a higher receptor binding and better internalization, with the consequence that tumour uptake of the tracer is intense. In vitro studies revealed that TATE, whether chelated with DTPA or DOTA, shows 14- to 17-fold higher affinity to sst2 than octreotide and 8- to 10-fold higher affinity than TOC. In studies performed by our group the favourable pharmacokinetic parameters of this tracer were shown [7]. Taking into account Polish experiences with the use of ^99m^Tc-EDDA/HYNIC-TATE, the focal lesions were better visualized by this tracer than by Octreoscan. It seems to be connected with higher affinity of [Tyr^3^]octreotate to sst2 and better special resolution due to ^99m^Tc physical properties. These results are consistent with the outcomes of preclinical studies which indicated that [Tyr^3^]octreotate radiotracers were characterized by the stated good sst2 affinity and high degree of internalization of this somatostatin analogue in tumour cells. Application of ^99m^Tc-EDDA/HYNIC-TATE SRS followed by radio-guided surgery (RGS) may significantly improve the rate of detection and efficacy of treatment of NETs of the gastrointestinal tract (GEP NET), especially in the presence of occult endocrine tumours. The imaging properties of ^99m^Tc-EDDA/HYNIC-TATE and possible 1-day imaging protocol creates potential for more common application of this tracer in oncology [8].

On the basis of available publications and Polish experiences since 2004, it can be concluded that ^99m^Tc-labelled somatostatin analogues essentially improved diagnostic efficacy and contribute to optimization of the neuroendocrine patients therapeutic procedure. The tracers are used for localization of sst-positive tumours and their metastases and above all as part of peptide receptor radionuclide therapy (PRRT) (qualification to ^90^Y, ^177^Lu or ^90^Y/^177^Lu radionuclide therapy and further control of therapy effectiveness) and “cold” somatostatin analogue therapy. Although it is expected that in future positron emission tomography (PET) examinations with the use of somatostatin analogues labelled with ^68^Ga will replace any other forms of sst-receptor positive tumour examinations, until then SRS with the use of analogues labelled with ^99m^Tc may be still considered a good alternative for the departments without PET scanners. From our point of view, ^99m^Tc-labelled somatostatin analogues are important and effective members of the radiolabelled peptide family.
